# Effect of the level of “*Candidatus* Liberibacter solanacearum” infection on the development of zebra chip disease in different potato genotypes at harvest and post storage

**DOI:** 10.1371/journal.pone.0231973

**Published:** 2020-04-28

**Authors:** Regina K. Cruzado, Mahnaz Rashidi, Nora Olsen, Richard G. Novy, Erik J. Wenninger, Nilsa A. Bosque-Pérez, Alexander V. Karasev, William J. Price, Arash Rashed

**Affiliations:** 1 Department of Entomology, Plant Pathology and Nematology, Aberdeen R&E Center, University of Idaho, Aberdeen, Idaho, United States of America; 2 Department of Plant Pathology, Citrus Research and Education Center, University of Florida, Lake Alfred, Florida, United States of America; 3 Department of Plant Sciences, Kimberly Research & Extension Center, University of Idaho, Kimberly, Idaho, United States of America; 4 Small Grains and Potato Germplasm Research Unit, United States Department of Agriculture, Agricultural Research Service, Aberdeen, Idaho, United States of America; 5 Department of Entomology, Plant Pathology and Nematology, Kimberly Research & Extension Center, University of Idaho, Kimberly, Idaho, United States of America; 6 Department of Entomology, Plant Pathology and Nematology, University of Idaho, Moscow, Idaho, United States of America; 7 Statistical Programs, College of Agricultural and Life Sciences, University of Idaho, Moscow, Idaho, United States of America; USDA-ARS Southeast Area, UNITED STATES

## Abstract

Potato psyllid (*Bactericera cockerelli* Sulc)-transmitted “*Candidatus* Liberibacter solanacearum” (Lso) has been negatively impacting the potato industry in the United States as well as other potato-producing countries. Lso has been linked to a condition known as zebra chip (ZC) that affects yield and quality of potato tubers. Efforts to find sources of resistance to ZC have primarily focused on greenhouse evaluations based on a single inoculation time prior to harvest. Plant response to infection, however, could be influenced by the developmental stage of the host plant, and ZC may continue to develop after harvest. The objectives of this study were to quantify Lso inoculation success, Lso titer, ZC severity and Lso development during storage in eight potato genotypes. These evaluations were conducted on plants infested with Lso-positive psyllids at 77, 12, and 4 days before vine removal (DBVR). The evaluated genotypes were categorized according to their relative resistance to Lso and tolerance to ZC symptoms. Lso inoculation success in the genotype family A07781, derived from *Solanum chacoense*, was lower than that of the susceptible control (‘Russet Burbank’). A07781-4LB and A07781-3LB genotypes were characterized relatively resistant to the pathogen and highly tolerant to ZC symptoms, while A07781-10LB was categorized as susceptible to Lso but relatively tolerant to symptom expression. In stored potatoes, increase in Lso concentrations was observed for all infestation times. However, significantly higher Lso titer was detected in tubers infested 12 DBVR and the effect was similar across genotypes. Overall, the A07781 family can be considered as a promising source of resistance or tolerance to ZC.

## Introduction

Zebra chip disease (ZC) represents an economic problem for the potato industry since it severely affects the yield and quality of production in the United States, Mexico, Central America and New Zealand [1–3]. ZC has been linked to the pathogen “*Candidatus* Liberibacter solanacearum” (Lso), a phloem-limited bacterium that is transmitted by the potato psyllid *Bactericera cockerelli* Sulc (Hemiptera: Triozidae) [2, 4]. ZC can potentially reduce potato yield and quality by 50%, and up to more than 85% in severely affected fields [5]. Consequently, the potato industry in certain areas has been compelled to increase the use of insecticides to reduce psyllid populations in an effort to control ZC spread [6, 7]. Frequent insecticide applications are costly [6–8] and controlling ZC can cause up to 55% reduction in growers’ returns because of the increase in input costs and reductions in yield and quality [6, 7].

Foliar ZC symptoms are characterized by upward rolling of the top leaves, purplish discoloration, shortened internodes, leaf-scorch, and the formation of aerial tubers, observed within three- or four-weeks following infection [9, 10]. The most characteristic symptom of ZC is brown discoloration and necrotic flecking of internal tuber tissue [11–13]. The severity of ZC symptoms in tubers has been shown to be positively correlated with variations in levels of certain phenolic compounds, reducing sugars and defense enzymes [14, 15]. Likewise, ZC symptom severity is known to be influenced by the infection time and host plant physiological responses [10, 16, 17]. Previous studies have shown that early-season infections produce severe symptoms in tubers whereas symptoms in late-season infections may not be visible in the tubers [15, 17, 18]. Recent studies have suggested that disease continues to impact tubers post-harvest, and during the process of storage [17, 19], a finding which is particularly relevant to the late-season infected potatoes where the tubers appear asymptomatic at the time of harvest [10, 15, 17, 18]. Lso acquisition success by potato psyllids may be affected by the distribution of Lso within the source plant, as it is known to be very heterogeneous [20, 21]. Inoculation success and disease development may also be affected by plant defense mechanism(s) activated by the pathogen [22, 23]. Therefore, it is expected that host plant genotypes differ in their degree of susceptibility to Lso and with respect to later disease development.

No effective integrated approach has been developed for the ZC pathosystem. As such, the potato industry has been forced to increase the use of insecticides to reduce populations of psyllids in an effort to curb ZC spread [6, 7, 24, 25]. The frequent use of insecticides, however, is unsustainable from both economic and insecticide resistant management standpoints [7, 8, 26–29], making development of an IPM strategy essential for management of this pathosystem [3, 30]. Host resistance is an effective component of IPM, and to date, a number of studies have screened potato genotypes for sources of resistance and/or tolerance against Lso, and/or potato psyllids, during vegetative developmental stages of the potato plant [31–34]. These studies have characterized resistance against ZC based on interactions between the insect and host plant [31, 32], or based on the evaluations of ZC symptom severity in fresh tubers at harvest [16, 33, 35, 36]. For instance, Butler et al. [31] reported reduced probing durations by potato psyllids on four of the evaluated potato genotypes in their study. Moreover, Rubio-Covarrubias et al. [33] reported limited ZC symptom expression (i.e., tolerance) in fresh tubers from selected potato genotypes. The observed tolerance in these genotypes was associated with reduced concentrations of phenolic compounds in tuber tissues [33]. None of these studies, however, considered the potential for differences in post-harvest disease development.

While tuber physiological responses to ZC generally appear to be correlated with symptom severity [14, 15], the expression of defensive responses to Lso infection are expected to vary among potato genotypes [35]. With the presence of such variability, the search for sources of resistance or tolerance to ZC is expected to result in identifying genotypes with relatively low susceptibility to Lso infection and/or potato psyllid infestation [37–39]. In relation to this, through a series of greenhouse screening trials, Diaz-Montano et al. [32] reported reduced fecundity of potato psyllids when they were exposed to selected potato genotypes derived from *Solanum etuberosum* Lindl. Further, Rashidi et al. [34] showed that clones derived from *Solanum chacoense* Bitter, identified as A07781-10LB, A07781-3LB, and A07781-4LB (Table 1) expressed lower susceptibility to ZC infection in fresh tubers at harvest when compared to other genotypes evaluated. Controlled conditions present in greenhouse experiments, however, can have an influence on the development of the disease as well as the success of Lso transmission [40]; field evaluations were needed to validate findings from greenhouse studies.

**Table 1:** Selected potato genotypes for greenhouse, field and storage study.

Genotype	Background	Reference
Russet Burbank	Susceptible control; US commercial cultivar	Munyaneza et al. 2011 [5]
A07781-4LB	Selected based on low ZC symptoms severity in the greenhouse experiment	Rashidi et al. 2017 [34]
A07781-3LB	Selected based on low ZC symptoms severity in the greenhouse experiment	Rashidi et al. 2017 [34]
A07781-10LB	Selected based on low ZC symptoms severity in the greenhouse experiment	Rashidi et al. 2017 [34]
A08399-6LB	Sri-Lankan cultivar HilStar in background	Unpublished data
PALB3016-6	Reduced oviposition; *S*. *guerreroense* and *S*. *chacoense* in pedigree	Unpublished data
A05379-211	Reduced growth index, reduced ZC severity in the greenhouse experiment; *S*. *etuberosum* and *S*. *berthaultii* in pedigree	Butler et al. 2011 [31], Diaz-Montano et al. 2013 [32]
Western Russet	Reported ZC tolerance/resistance in New Zealand	Unpublished data

The present study was set up to evaluate the inoculation success and variations in susceptibility to ZC (i.e., Lso infection and symptom expression) among eight potato genotypes. The potato genotypes were first evaluated for Lso inoculation success via greenhouse assays. In addition to the greenhouse evaluation, this study quantified and compared genotypes for ZC development that occurs both early and late in the growing season in the field. This last approach allowed identification of potential variation in relative susceptibility of the evaluated genotypes to Lso infection at different stages of plant development [15]. This question is applicable to naturally occurring field infections which can happen throughout the growing season [41]. In the US Pacific Northwest, the infective psyllids have been found near potato fields during the month of July [42], although lower levels of Lso-positive psyllids could be also found later into the summer [43, 44]. Resistance or tolerance to ZC was analyzed based on their relative susceptibility to the pathogen and tolerance to ZC symptom expression of tubers [34, 40]. Moreover, we continued quantifying Lso development post-harvest for the duration of storage to determine whether changes in the pathogen concentration during storage vary among potato genotypes.

## Materials and methods

### Plant and insect material

Eight potato genotypes (*Solanum tuberosum* L.) provided by the USDA-ARS, Small Grains and Potato Germplasm Research Unit, Aberdeen, Idaho, were selected for this experiment. Seven genotypes had previously been reported as tolerant or resistant to ZC and/or the potato psyllid [31, 32, 34]. The variety Russet Burbank was included as the susceptible control (Table 1).

Lso-positive and Lso-negative colonies of Central haplotype potato psyllid [45] were both used for the greenhouse study whereas psyllids from the infected colony only were used for the field study. The haplotype of the potato psyllid was confirmed by polymerase chain reaction (PCR) and followed by a digestion with restriction endonucleases, as described by Swisher and Crosslin [46]. Lso positive colonies of the psyllids were confirmed to contain Lso B haplotype, following the procedure described by Wen et al. [47]. Lso-positive and negative colonies were reared on potato (var. Russet Burbank) and maintained in climate-controlled growth chambers [18–27°C; 16:8 hrs photoperiod (Light: Dark)] in 60 x 60 x 60 cm bugdorm cages (BioQuip Products, Rancho Dominguez, CA). Prior to each inoculation (see below), Lso incidence in colonies was confirmed by testing 10 individuals from each colony, following Crosslin et al. [48]. The Lso incidence in the colonies ranged between 90 and 100 percent in both years of the study.

### Greenhouse assay: Liberibacter inoculation success

Inoculation success assay was conducted in greenhouses at the University of Idaho, Aberdeen Research and Extension Center, Aberdeen, ID, from February through July 2016. Tubers from eight genotypes were planted in 7.57-liter pots containing a mixture of 70% sand, 20% peat moss (Sun Gro Horticulture Canada Ltd., Seba Beach, AB, Canada), 10% vermiculite (Therm-o-Rock West, Inc., Chandler, AZ, USA) and fertilizer (Osmocote, Nitrogen/Phosphorous/Potassium mix of 14/14/14; Scott-Sierra Horticultural Products Co., Marysville, OH, USA). Plants were maintained in controlled greenhouse conditions with temperatures ranging between 16 (night) and 23 (day)°C, on a 12:12 hrs photoperiod (Light: Dark). The light was artificially provided by six sets of 432-Watt Sun Blaze T5 48” 8 Fluorescent Lamps (Sunlight Supply ®, Inc. Vancouver, WA, USA) during 12 hours per day.

Plants were arranged in a completely randomized design and the experiment was repeated twice with each block timing planted one week apart, in two separate greenhouses. For each inoculation time, there were 12 plant-replicates per genotype and two non-infected control plants. One of the control plants was not infested with potato psyllids (no-psyllid control), while the other control plant was infested with potato psyllids from a Lso-negative colony. No tubers from either control group tested positive for Lso over the course of the experiments. Plants were inoculated with three psyllids two weeks after plants reached 80% of total emergence. For inoculations, one frame-less leaf clip cage (BioQuip Products, Rancho Dominguez, CA) 2.54-cm in diameter was installed on a single leaflet of a fully expanded middle leaf of each plant. Three Lso-positive psyllids were collected from the colony using an aspirator and released into each leaf cage. For control plants infested with non-infective psyllids, three Lso-negative psyllids were released into each leaf cage. All plants were covered individually with a mesh bag for the 48 hours of inoculation access period (IAP).

After IAP, mesh bags were removed, psyllids from each plant were collected and placed into a 2 ml microcentrifuge tube. The psyllids (composite sample of three psyllids) were stored at -20°C until Lso quantification by quantitative polymerase chain reaction (qPCR) as described below. Plants were sprayed with insecticide immediately, and also one week after psyllids removal, to eliminate possible presence of early developmental stages of the potato psyllids (i.e., nypmhs from hatched eggs laid during the IAP). The tank mix consisted of 0.70 ml/L of Warrior II (248 g/L of lambda-cyhalothrin [a.i], 1.9x10^-6^ g of a.i per plant) (FMC Corporation, GA, USA) and 1.95 ml/L of Movento (480 g/L of spirotetramat [a.i], 0.043 g of a.i per plant) (Bayer CropScience, NC, USA). These sprays were repeated one week after psyllid removal. Plants were maintained in the greenhouse for approximately 10 weeks, after which potato vines were removed by cutting the above ground stem at the very base (‘vine removal’). Tubers were harvested two weeks after vine removal. Tubers less than 2 cm in diameter were discarded. From the remaining tubers of each plant, four were randomly selected for Lso analysis to determine inoculation success. The selected tubers were sampled at the stolon attachment end by removing 100 mg of tissue using a 6-mm Harris UNI—CORE^TM^ (GE Healthcare Life Sciences, Buckinghamshire, UK). Samples of tubers were stored in -20°C for DNA extraction and analysis of Lso status by qPCR.

### DNA extraction and Lso quantification

Total DNA from tuber samples and psyllids (composite sample of 3 psyllids) were extracted using the CTAB (hexadecyltrimethyllammonium bromide) method. DNA extraction of tubers was performed following Rashidi et al. [34] whereas DNA from psyllids was extracted according to Marzachi et al. [49]. DNA was quantified (absolute quantification) by qPCR using SYBR Green in a CFX Real-Time PCR System (BioRad Laboratories, Hercules, CA, USA). The qPCR reaction contained 150 nM of each of the primers, HLBr and LsoF [50, 51], 1X SsoAdvanced Universal SYBR Green Supermix (BioRad Laboratories, Hercules, CA, USA), and 1 μl of DNA template. The amplification program was set at one cycle at 98˚C for 2 min, 40 cycles of 95˚C for 10 sec, 62˚C for 20 sec, which was followed by a melt curve (65˚C to 95˚C, 0.5˚Cs^-1^ increments). To estimate copy numbers, plasmid (pIDTSMART–KAN) containing a known copy number of Lso was used to build the standard curve based on eight 10-fold serial dilutions [20]. Negative controls including DNA from healthy plants and water (no template control) were also included in all qPCR analyses.

### Field evaluations: Relative susceptibility to Lso infection and tolerance to ZC

Field experiments were conducted in 2016 and 2017 at the University of Idaho, Kimberly Research and Extension Center, Kimberly, ID. Seed potato pieces were planted on 5 May 2016 and 3 May 2017. Potato vines were removed on 6 September 2016 and 5 September 2017. Tubers were harvested following a 14-day skin-set period on 20 September 2016 and 18 September 2017.

### Study design

Treatments were laid out in a randomized complete block design, with 10 blocks in 2016, and 8 blocks in 2017. Each block included four cages, each infested at either 77, 12 or 4 days before vine removal (DBVR). One cage per block was not infested with the potato psyllids and was used as a non-inoculated control. One seed potato from each genotype was planted randomly inside each cage (8 seeds/cage). Seeds were planted on two rows of four seeds with 30-cm within-row spacing and 91-cm spacing between rows. Cages were built like hoop houses and were covered with 1.5 x 2.4 x 1-m (W x L x H) 4750 plastic mesh (U.S. Global Resources, Seattle, WA) over SunGUARD® II fiberglass rods (Geoteck Inc., Stewartville, MN).

The first inoculations were initiated three weeks after plants reached 80% of total emergence for all genotypes (approximately 46 days after planting). For inoculation, five Lso-positive psyllids were released at the base of each individual plant (40 psyllids per cage). Plants were exposed to Lso-positive psyllids for an IAP of 7 days, except for the last infestation (4 DBVR), which had an IAP of 4 days. Cages infested at 77 DBVR and 12 DBVR were sprayed twice and once, respectively, one and two weeks after inoculation, with a mix of 2.11 ml/L of Movento (480 g/L of Spirotetramat [a.i]) (Bayer CropScience LP, NC) and 1.58 ml/L of Agri-Mek (22 g/L of Abamectin [a.i]) (Syngenta Crop Protection, NC). At the end of the season, the vines were removed 129 days after planting (‘vine removal’). The removal of the foliar tissue was performed mechanically by cutting vines with pruning shears at the very base. Tubers were harvested 2 weeks after vine removal and stored at 12.7°C (95% RH) and evaluations were performed within 3 days after harvest.

### Evaluation of ZC symptoms severity and Lso quantification in tubers

After harvest, four tubers per plant were randomly selected for evaluations of ZC symptom severity and Lso quantity at harvest. Tubers less than 2 cm in size were not included in the study. ZC symptoms were evaluated by removing a thin slice at the tuber stem end (stolon attachment end). ZC severity was scored on a categorical scale of 0 to 3, according to Rashed et al. [15]; score “0” indicated asymptomatic tubers whereas score “3” was indicative of severe tuber symptoms. For Lso quantification, 100 mg of tissue was removed from the stem end of each tuber, using a 6 mm Harris UNI-CORE^TM^ (GE Healthcare Life Sciences, Buckinghamshire, UK). Samples were stored in -20°C for DNA extraction and Lso quantification. DNA extraction was performed by the CTAB method described by Rashidi et al. [34]. DNA extraction and Lso quantification by Real-time PCR (qPCR) were performed as previously described. All sampled tubers underwent the storage process after the at-harvest scoring/sampling.

### Lso susceptibility and ZC symptom severity in evaluated genotypes

To categorize relative susceptibility and tolerance of genotypes, pathogen titer (Y = Lso titer) and ZC symptom severity score (X = symptom score) were used to construct a two-dimensional graph area that was divided into four quadrants [34]. Each quadrant was defined as a category of relative susceptibility to the pathogen and tolerance to the infection. The evaluated genotypes were placed into a scatter plot graph according to their average Lso titers and symptom severity scores. Quadrants were delimited by averages of both Lso titers and ZC symptom severity score across all genotypes in the study. Genotypes were categorized as relatively susceptible or low susceptibility to Lso, and relatively tolerant or intolerant based on ZC symptom expression. High susceptibility is defined as the inability of a plant to stop or reduce the development of the pathogen [52]. Tolerance refers to the ability of a plant to limit symptom expression, regardless of the level of pathogen multiplication [52].

### Lso development in potato genotypes during storage

Following at-harvest evaluations, potato tubers were stored at the University of Idaho, Potato Storage Research Facility at Kimberly R&E Center, Kimberly, ID. Tubers from each plant were placed inside plastic mesh bags. Crates containing the bags were stored under the following conditions: 1) 12.7°C for two weeks (healing period), 2) temperature ramp-down to 8.9°C at 0.3°C per day within approximately 2 weeks, and 3) holding temperature of 8.9°C for approximately 21 weeks. Relative humidity was maintained at 95% during the different stages of storage and sprouting was inhibited by applying 22 ppm of chlorpropham (CIPC; Decco, Elf Atochem North America, Monrovia, CA) 67 days after harvest. Tubers evaluated at harvest were re-evaluated for Lso titer at the end of storage. Changes in Lso titer during storage were compared among the evaluated genotypes since changes in symptom severity might have been influenced by the continued impact of phenolics on tuber tissue during storage. Lso titers were quantified as described previously. Changes in titer were calculated by subtracting the initial at-harvest Lso titers (Lso (χ_ref_)) from those of post storage (Lso (χ)) divided by the initial quantity (Lso (χ_ref_)), where Lso (χ, χ_ref_) = [(Lso (χ)—Lso (χ_ref_)]/Lso (χ_ref_).

### Statistical analysis

#### Greenhouse evaluation

A generalized linear mixed model (GLMM) assuming a binomial distribution was used to evaluate the Lso infection success in tubers [53]. The model included block, potato genotype, the Lso titer of psyllids used for Lso inoculations (i.e., psyllid titer load), and block-genotype interaction as interaction term as fixed factors and plant replicates as a random factor. Factors with non-significant (P > 0.05) effect were removed from the model, in a stepwise approach to improve the sensitivity of our model to detect differences among genotypes.

#### Field and storage evaluations

A GLMM assuming a lognormal distribution was used to compare Lso titers and ZC symptom severities among potato genotypes. For each plant, data for Lso quantity and ZC symptom severity were determined based on average titer and ZC score in the four randomly selected tubers. A fixed value of 1.5 was added to all Lso concentrations to allow log transformations where zeros were present. Similarly, a value of 0.1 was added to averages of ZC symptoms severity scores prior to the analysis. For Lso titer and ZC symptoms severity, the model included year, genotype, time of infestation, two-way interactions, and the three-way interaction terms as fixed factors. Since both Lso titer and ZC symptom analysis were pooled over years, block and block-treatment interaction within year were considered as random factors. Following analysis, Lso titer and ZC symptoms were compared between Russet Burbank and each of the other genotypes using pairwise t-test comparisons. A similar model and approach were used in the analysis of changes in Lso titer during storage. Unless otherwise noted, reported mean values and associated summary statistics for all responses are untransformed values based on model estimates. All analyses were carried out with SPSS (IBM, ver. 24.0).

## Results

### Greenhouse assays: Lso inoculation success

**Fig 1:**
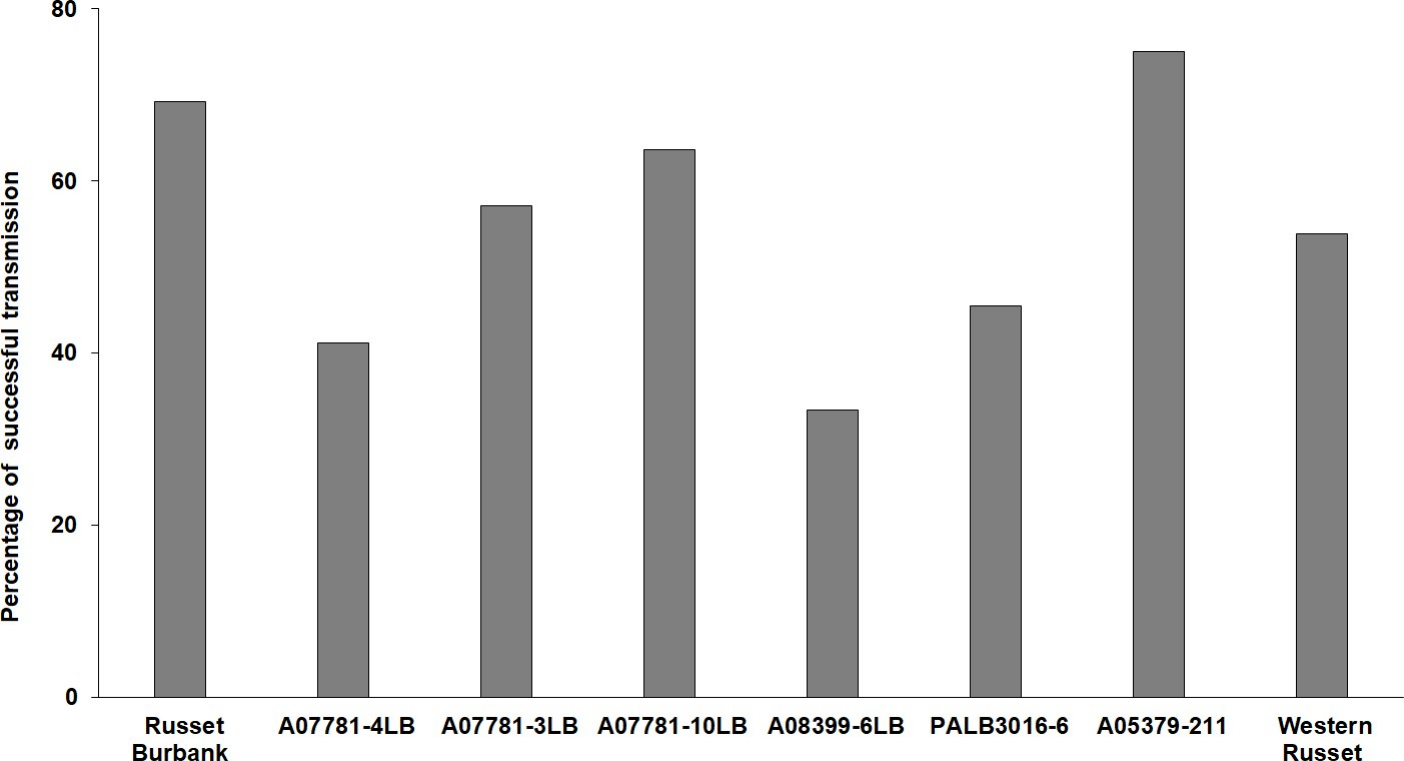
Percentage of plants infected with “*Candidatus* Liberibacter solanacearum” after inoculation with Lso-positive potato psyllids. The experiment was conducted in 2016 and Lso infection was confirmed by testing fresh tubers of each genotype.

There was no significant difference in the successful Lso inoculation among the evaluated genotypes (F_*7*,*87*_ = 0.69; *P* = 0.68). The Lso inoculation percentages ranged between 33% (A08399-6LB) and 75% (A05379-211) (Fig 1). While not significant overall, genotypes A07781-4LB, A08399-6LB and PALB3016-6 tended to show lower inoculation success than the susceptible control and were below the 57% overall average. Russet Burbank (77%) and A05379-211 (76%) were the two genotypes with relatively higher rates of inoculation success (Fig 1).

### Field evaluations: Relative susceptibility to Lso infection and tolerance to ZC

**Fig 2:**
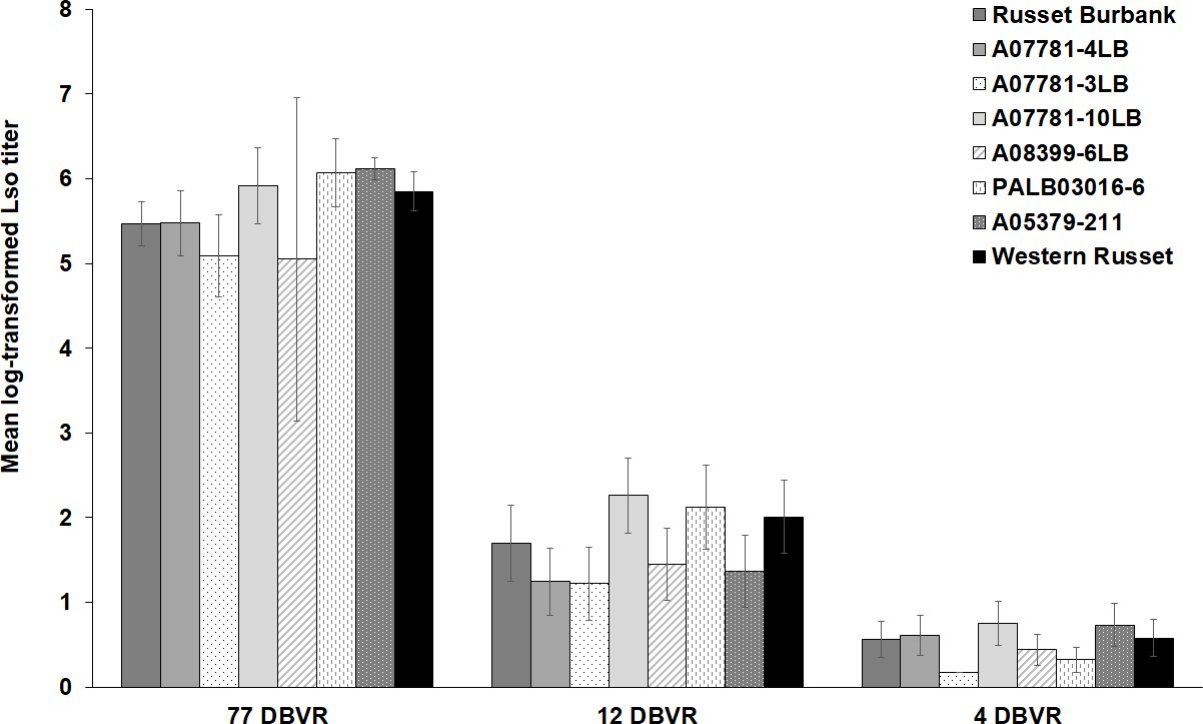
Mean log-transformed Lso titer in potato genotypes infested at 77, 12 or 4 days before vine removal (DBVR). Lso quantity was analyzed in tuber samples at harvest. Russet Burbank was used as the susceptible control. Error bars represent standard error (±1).

Lso titer was not significantly affected by the genotype-infestation time interaction (F_*14*,*381*_ = 0.71; *P* = 0.769) nor by the genotype-year (F_*7*,*381*_ = 0.28; *P* = 0.959), year-time of infestation (F_*2*,*381*_ = 0.54; *P* = 0.585), genotype-year-time of infestation (F_*14*,*381*_ = 0.66; *P* = 0.810) interactions, year (F_*1*,*381*_ = 1.87; *P* = 0.171), or genotype (F_*7*,*381*_ = 2.01; *P* = 0.053) (Fig 2). However, titer of Lso was significantly influenced by the time of infestation (F_*2*,*381*_ = 325.96; *P* < 0.001). Tubers infected early in the season (77 DBVR) had significantly higher Lso titer, whereas the lowest Lso titer was detected in tubers infected at 4 DBVR (Fig 2). The pairwise comparison of Lso titers among time of infestations showed that Lso titers were significantly different between 77 and 12 DBVR (*P* < 0.001) as well as 12 and 4 DBVR (*P* < 0.001).

**Fig 3:**
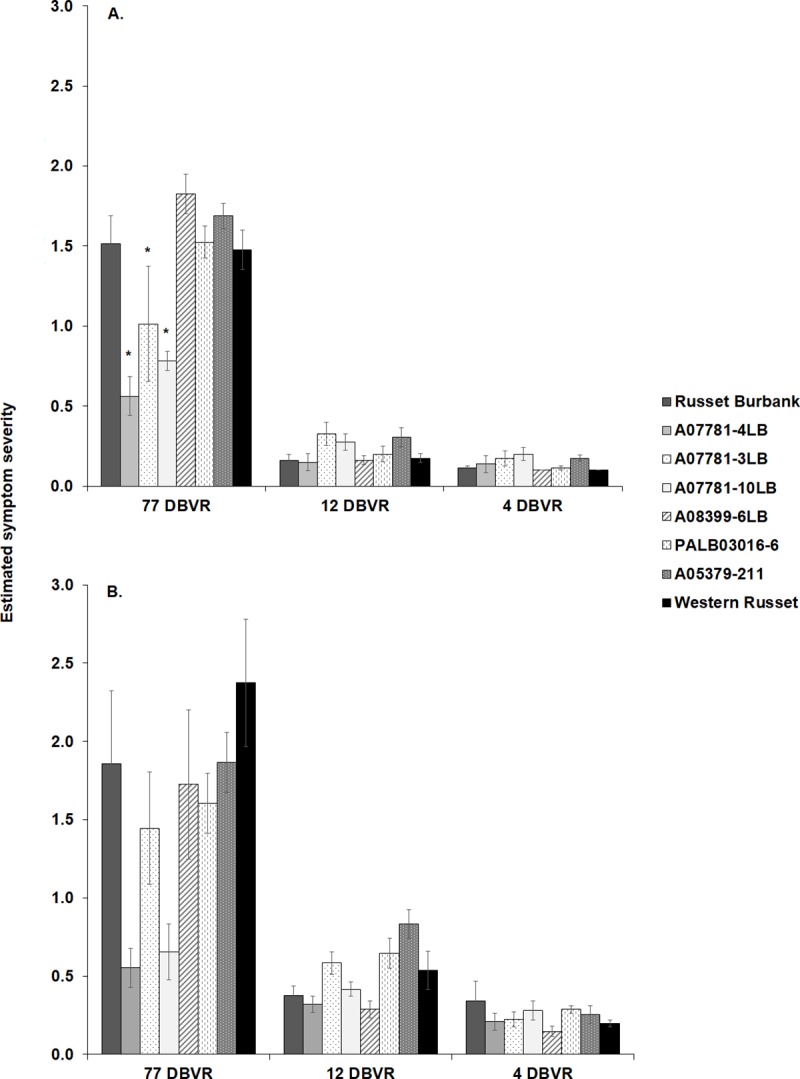
ZC symptoms severity in potato genotypes infested at either 77, 12 or 4 DBVR. Evaluations were conducted in tubers at harvest in (A) 2016 and (B) 2017. Significant differences in ZC symptom severity scores among genotypes in each time of infestation are indicated by asterisks. The severity of ZC symptoms in A07781-3LB, A07781-4LB, and A07781-10LB were significantly lower than other genotypes at 77 DBVR in 2016. Statistical analysis was performed on log-transformed data. Error bars represent standard error (±1).

For ZC symptoms, a significant difference in the severity of symptoms was detected between the year-time of infestation interaction (F_*2*,*381*_ = 25.80; *P* < 0.001) as well as between the two years (F_*1*,*381*_ = 18.58; *P* < 0.001) of the study with a greater average symptom score observed in 2017. Thus, among-genotype symptom analysis was conducted separately for each year (Fig 3). Nonetheless, overall ZC symptom severities were significantly affected by the time of infection in both years of the study (F_*2*,*381*_ = 379.77; *P* < 0.001), with the highest and the lowest symptom severities scored in the 77 and 4 DBVR, respectively (Fig 3). The pairwise comparison of ZC symptoms among time of infestations showed that severities of ZC symptoms were significantly different between 77 and 12 DBVR (*P* < 0.001) as well as 12 and 4 DBVR (*P* < 0.001).

The separate analysis for each year showed a significant genotype-time of infestation interaction in 2016 (F_*14*, *216*_ = 5.35; *P* < 0.001) but not in 2017 (F_*14*, *165*_ = 1.71; *P* = 0.057). The genotypes A07781-4LB (*P* < 0.001), A07781-3LB (*P* = 0.007), and A07781-10LB (*P* < 0.001) expressed significantly lower symptom severity than the other genotypes in 2016 at the 77 DBVR treatment (Fig 3). Albeit nonsignificant, A07781-4LB and A07781-10LB showed nominally lower expression of symptoms than the other genotypes in 2017 at the 77 DBVR time of infestation (Fig 3). Symptom severity of A08399-6LB (*P* = 0.005) was significantly higher in comparison to Russet Burbank in 2016. A different pattern was observed within the 12 DBVR treatment as symptom severities tended to be higher than the susceptible control especially in A07781-3LB and A07781-10LB whereas the symptom severity of A07781-4LB tended to be lower in both years (Fig 3). Also, ZC severity score was significantly affected by time of infestation (2016: F_*2*, *216*_ = 170.25; *P* < 0.001; 2017: F_*2*, *165*_ = 70.20; *P* < 0.001) and by potato genotype (2016: F_*7*, *216*_ = 5.85; *P* < 0.001; 2017: F_*7*, *165*_ = 5.89; *P* < 0.001) (Fig 3). ZC symptom scores and Lso titer were low with few tubers exhibiting symptoms in tubers infested 12 and 4 DBVR in either 2016 or 2017 (Fig 3). Thus, comparison of symptom severity for the analysis of relative susceptibility was examined in plants which were infested 77 DBVR.

**Fig 4:**
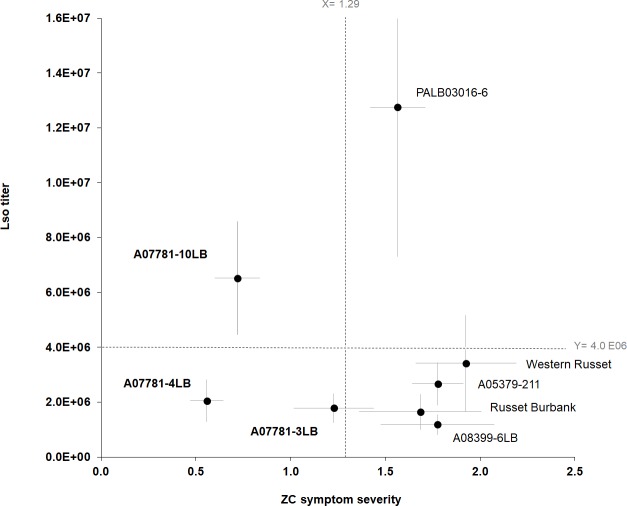
Relationship between Lso titer and ZC symptoms severity of potato genotypes infested at 77 DBVR. Assessments were made in tuber samples at harvest from season 2016 and 2017. Russet Burbank: susceptible control. Two sets of dotted lines represent overall averages of symptom severity and Lso titer across the eight genotypes. Error bars represent standard error (±1).

Relative resistance to Lso and tolerance to ZC symptoms were examined among the eight evaluated genotypes by plotting the relationships between pathogen quantity and symptom severity in freshly cut tubers from the early infestation (77 DBVR). Since the interaction between year and potato genotype was not significant for Lso quantity and because symptom expression in 2016 and 2017 followed a consistent pattern (Fig 3), the data for 2016 and 2017 were pooled for plotting purposes (Fig 4). The three siblings belonging to the A07781 family were categorized as either resistant or tolerant in relation to other evaluated genotypes. Relatively low Lso titer and mild ZC expression resulted in A07781-4LB and A07781-3LB being categorized as relatively resistant genotypes, while relatively high Lso titer but mild symptom expression identified A07781-10LB as a tolerant genotype. The susceptible control Russet Burbank, the commercial variety Western Russet, and breeding clones A05379-211 and A08399-6LB were categorized as intolerant. Although they had relatively low titer levels, they still expressed relatively severe ZC symptoms. In addition, PALB03016-6 showed relatively higher symptom severity scores and Lso quantities leading to be categorized as susceptible.

### Lso development in potato genotypes during storage

**Fig 5:**
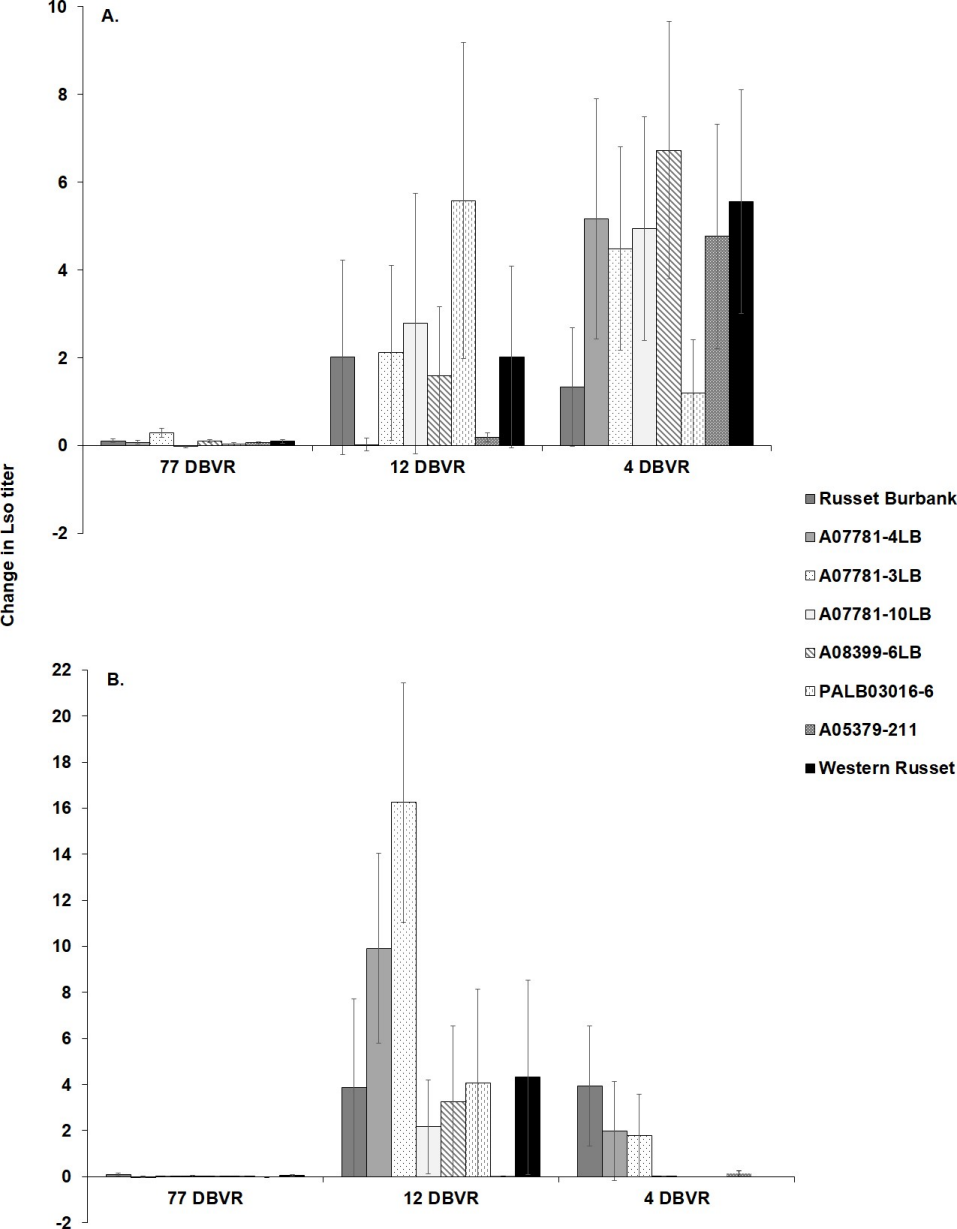
Changes in Lso titer in tubers of potato genotypes between harvest and storage. Tubers from (A) 2016 and (B) 2017 were infested at either 77, 12 or 4 DBVR and were evaluated for their Lso titer at harvest and after cold storage. Although, no significant differences were detected in time of infestation-genotype interaction in both 2016 and 2017, the Lso change in A07781-3LB tended to be higher than the susceptible control, Russet Burbank in 2017. Bars represent estimated values of Lso change. The change in Lso titer was estimated from log-transformed Lso copy numbers. Error bars represent standard error (±1).

Due to a significant effect of the year-time of infestation interaction, changes in Lso titer were analyzed separately for each year following a similar approach described in the prior harvest analyses (Fig 5).

The change in Lso titer was not significantly influenced by time of infestation-genotype interaction (2016: F_*14*, *212*_ = 0.98; *P* = 0.472; 2017: F_*14*, *163*_ = 1.69; *P* = 0.061) in either year. In both years the shift in Lso titer was significantly affected by the time of infestation (2016: F_*2*, *212*_ = 6.60; *P* = 0.002; 2017: F_*2*, *163*_ = 10.32; *P* < 0.001). The change in Lso titer in the 77 DBVR treatment was lower than both 12 and 4 DBVR treatments in 2016. Change in Lso titer was significantly affected by the potato genotype across time of infestations in 2017 (F_*7*, *163*_ = 2.26; *P* = 0.032) but not in 2016 (F_*7*, *212*_ = 6.60; *P* = 0.950).

Additionally, the pairwise comparisons of change in Lso titer among the three time of infestations showed significant differences between 77 DBVR and 4 DBVR (*P* = 0.001), but not between 77 DBVR and 12 DBVR, or between 12 and 4 DBVR in 2016. On the other hand, the change in Lso titer was significantly different between 77 DBVR and 12 DBVR (*P* < 0.001) as well as between 12 DBVR and 4 DBVR (*P* = 0.001) but not between 77 DBVR and 4 DBVR in 2017. Also, the comparison of Lso change between Russet Burbank and other genotypes showed that Lso titer in A07781-3LB tended to be higher than Russet Burbank in 2017 in the 12 DBVR treatment.

## Discussion

In the present study we evaluated and compared inoculation success of Lso in the greenhouse and relative susceptibility to ZC for infections occurring at different times during the field season among eight potato genotypes. Moreover, for the first time, screening assays were extended beyond harvest and into storage where changes in Lso titer were also compared among the evaluated genotypes. Despite originality in assessing host susceptibility both early and late into the growing season, our field study also helped to validate findings from an earlier greenhouse study, which reported relative resistance and tolerance in three genotypes, all belonging to the A07781 family [34]. Overall, the A07781 siblings, derived from *Solanum chacoense* re-emerged as a promising group in the field evaluations, suggesting this germplasm will be helpful in breeding programs as a source for developing commercially acceptable varieties with reduced susceptibility to ZC disease.

A07781-4LB, A07781-3LB, A07781-10LB, A05379-211 and Russet Burbank were the five genotypes shared between our study and the greenhouse study by Rashidi et al. [34]. Those five genotypes all showed inoculation success rates of < 37% in the greenhouse [34], here however, except for A07781-4LB, the remaining four genotypes had inoculation success rates exceeding 50%. The higher rate of inoculation success in the present study was most likely because of the higher number of Lso-positive psyllids used for inoculations. It is known that the likelihood of successful transmission is enhanced as the number of potato psyllid vectors increases [40]. We chose to increase the number of psyllids for the inoculation experiment by one to assure survival of more than one psyllid on each plant after IAP, thus increasing the number of usable data points in our inoculation assay. Nonetheless, similar to [34], variation in inoculation success among genotypes was not statistically significant.

Potato psyllid infestations may occur at different times throughout the season in a pattern that appears to be location specific. For example, early-season infections are common in southern states because psyllids are present the whole year and they move early into potato fields. Conversely, in Idaho, late-season infections are likely to occur [41], a pattern that may correspond to the increase in the number of potato psyllids captured in the field late in the season [44]. Time of infection is known to affect both the severity of ZC symptoms [17, 18] and the Lso quantity within host plants. Likewise, our results showed that ZC symptom severity and Lso titer vary with the time of infestation (or infection). Overall, the highest ZC severity scores and Lso titers were associated with early-season infections whereas late-season infections had the lowest ZC severities and Lso titers. In spite of this overall pattern, both Lso titers and severity of ZC symptoms were greater in 2017 compared to 2016. Since both potato psyllids and Lso are known to be sensitive to temperature increase [3, 24, 54], variations in temperature was initially considered as a potential explanation for the observed between-year differences. The average daily maximum temperatures, for the duration of our study, however, did not drastically differ between the two years (2016: 36.6°C; 2017: 37.2°C) (https://www.usbr.gov/pn/agrimet/). Potential difference in the quality of vectors, between the two years, was another factor that might have impacted the amount of the initial inoculated inoculum, subsequently impacting ZC development [40]. Moreover, plant response to infection may also vary at different stages of plant development [15].

At 77 DBVR, genotypes A07781-4LB and A07781-3LB showed low susceptibility to Lso and reduced ZC symptoms relative to other evaluated genotypes. A07781-10LB appeared susceptible to Lso but tolerant to ZC symptoms, meaning that ZC symptom expression was reduced despite high Lso titers in tubers of this clone. Findings from the field evaluations were in accordance with our previous greenhouse reports [34]. Russet Burbank, Western Russet, A05379-211, A08399-6LB were categorized as relatively intolerant to ZC, since they expressed symptoms higher than those observed in the A07781 family. Although symptom expression was limited in the A07781 family at 77 DBVR, two of the three genotypes from this family, A07781-3LB and A07781-10LB, appeared relatively more intolerant compared to the Russet Burbank control, or the other commercial variety, Western Russet, in the 12 DBVR infestation treatment. This suggested that the degree of resistance to ZC is likely influenced by the time of infection (i.e., plant developmental stage). This variation in susceptibility over time has also been demonstrated in other vector-borne disease systems [55–57].

Our failure to detect statistical differences in Lso titers among genotypes was likely due to the high degree of variation in Liberibacter titer within plant tissue as seen in other similar pathosystems [55, 56]. Lso is known to be heterogeneously distributed within plant tissues [20, 21], even among tubers of the same plant, because Lso movement inside phloem is driven by the vascular architecture of potato plants [21]. The “biological” variations (*P* = 0.051) in Lso titers among evaluated genotypes must not be overlooked, simply for surpassing an arbitrary cut-off value of *P* ≦ 0.05, particularly where consistency in pattern persists between studies.

At 4 DBVR, symptoms were not detectable at harvest. Although successful Lso translocation from leaves to tubers has been reported in potato plants infected 4 days before harvest [19], ZC symptom may not be observed for infestations that occur up to one week before harvest [15, 17]. Lso detection success may also be limited in the late infestation times of 4 and 12 days before vine removal, as pathogen quantities are at undetectable levels; many tubers that are infected late in the season may proceed to test positive after a few months of storage [19]. Although the IAP was shorter in the 4 DBVR than 12 and 77 DBVR, because vines were removed only four days after infestation, it had minimal effect on inoculation rate as evident form our post-storage Lso detection success. Adults of the potato psyllids are efficient in transmitting Lso with a transmission success rate of up to 100% for multiple psyllids, within 48 hours of IAP [40].

Western Russet, A05379-211, and A08399-6LB had relatively lower Lso titers but were highly intolerant of ZC as they expressed relatively higher ZC symptom severity. PALB3016-6 was considered as a Lso-susceptible genotype and highly intolerant with respect to symptom expression. In this study, Russet Burbank (susceptible control) showed relatively low susceptibility to Lso but it was highly intolerant of infection due to high levels of symptom expression. Tolerance can result from a decrease in the intensity of host physiological responses to Lso infection. This response can be expressed as reduced production of phenolic compounds, known to be associated with ZC symptom expression and have previously been documented in tolerant potato clones [14, 15, 16].

The development of Lso during storage was also reflected in the significantly higher number of Lso-positive tubers after storage compared to those at harvest, which was in line with our findings from a previous storage study [19]. The post-storage evaluations also revealed that the change in Lso titer during storage was greater in tubers from plants which were infested 12 DBVR before vine removal, compared to those of 77 or 4 DBVR. Although the highest Lso titer was detected in 77 DBVR at harvest, the further development of Lso during storage was mostly reduced because the development of Lso already reached its limit at harvest. This pattern is consistent with Wenninger et al. [17] in which, although Lso titer were not assessed, the largest increase in disease symptom severities between harvest and storage were observed in inoculations that occurred around 2–3 weeks before vine kill; earlier infestations seem to have plenty of time to develop in tubers before harvest and, therefore, strong differences between harvest and storage ratings may not be present. Undetectable levels of Lso titer in tubers from the 4 DBVR treatment was indicative of extremely low Lso titer. In this infestation treatment, the relatively lower Lso titer after storage (post-storage) which was due to the lower Lso concentrations at harvest may have contributed to the failure in detecting significant increase in Lso titer during storage.

The Lso development, measured as change in titer between harvest and the end of storage, revealed significant variation among genotypes in the 12 DBVR treatment only. For example, the susceptible and intolerant genotype PALB3016-6 had a significantly higher shift in Lso titer in 2016 whereas the relatively less susceptible, yet tolerant, genotype A07781-3LB showed a higher increase in Lso titer in 2017. Therefore, the change in Lso during storage may not offer a reliable measure of susceptibility to Lso.

It is important to note that Lso titer in potato can vary with host genotype, the holding temperature, and the stage of storage [36]. Our study only quantified the change of Lso between harvest and the end of the storage. For this purpose, pre-harvest tissue sampling was needed. Tissue damage, however, may be problematic since polyphenol oxidases are known to become activated in response to mechanical damage facilitating the oxidation of monophenols to polyphenols that are known to be toxic to pathogens [58, 59]. In this study we assumed a similar response across genotypes to the tissue samplings performed at harvest; Lso development during storage could have been altered to some extent by wound-induced activation of defense responses before storage [24, 59]. To address this possibility, this study also included a subset of tubers that were kept intact until after harvest as controls for the potential effect of pre-storage sampling; no such an effect was detected (data not presented).

In our study, A07781 siblings expressed relative resistance or tolerance to ZC, which is consistent with previous findings for the A07781 clones in other greenhouse studies [34]. A07781-3LB and A07781-4LB were categorized as relatively resistant to Lso and highly tolerant of ZC symptoms, and A07781-10LB was considered susceptible to Lso but tolerant to the expression of ZC symptoms. Although the time of infection affected the expression of symptoms and susceptibility of most of genotypes, A07781-4LB appeared tolerant across the three times of infestation and less-susceptible to Lso at 77 and 12 DBVR.

In summary, A07781 siblings should be further considered as sources of resistance and tolerance genes to ZC in future breeding. Results from our storage study suggest that Lso continues to multiply and affect tubers during storage, regardless of the host genotype. Finally, we would like to reemphasize that susceptibility to Lso and tolerance to symptom expression are both relative terms, influenced by the pool of genotypes that are being evaluated within a given environment at any given time. Plant response to Lso infection can be influenced by the time of infection, highlighting the importance of ecological studies to determine vector/pathogen arrival with respect to host crop phenology within regions.
